# Exploring the relationship of cognitive style with psychosocial factors in Kosovo youth

**DOI:** 10.1192/j.eurpsy.2025.1817

**Published:** 2025-08-26

**Authors:** S. Mustafa, N. Fanaj, E. Krasniqi

**Affiliations:** 1 AAB College, Prishtina; 2 PMSH, Prizren; 3 Alma Mater Europae Campus College Rezonanca; 4 UBT College, Prishtina, Kosovo

## Abstract

**Introduction:**

Recently it was highlighted that individuals not only vary in their beliefs but also in the manner of their thinking, and this diversity can significantly predict various crucial psychological outcomes.

**Objectives:**

This research addresses the underexplored relationship between cognitive styles and psychosocial factors like hope, subjective well-being, coping and social support, specifically in Kosovo. These variables have not been investigated in Kosovo before, making this study the first of its kind.

**Methods:**

Its cross-sectional study. The sample consisted of 490 students aged 15 to 23 (Mage=19.06; SD=4.17). Participants completed the instruments below: Adult Hope Scale ; Brief COPE Scale ; Oslo 3-item Social Support Scale; WHO-5 Well-Being Index ; Rosenberg Self-Esteem Scale and Cognitive Reflection Test . Data processing was done with SPSS 27.0 and Microsoft Excel 2019.

**Results:**

Significant negative correlations were observed between Cognitive Reflection Test scores and gender (r = -.197, p < 0.00), Adult Hope Scale score (r = -.173, p < 0.00), WHO-5 Well-being Index (r = -.098, p < 0.04) and Emotion–Focused Coping (r = -.125, p < 0.01). However, no significant correlations were found with Social Support, Problem-Focused-Coping, or Dysfunctional Coping (all p > 0.05). The correlational analysis, suggested that individuals with higher analytical thinking tendencies are more likely to be male, have lower levels of hope, employ less emotion-focused coping strategies and reported lower subjective well-being.

**Conclusions:**

The study emphasized that psychosocial factors are intricate, influenced by diverse elements. While cognitive abilities, as measured by the Cognitive Reflection Test, played a role in some aspects of these factors, they didn’t fully explain their complexity. Therefore, the research suggests that further investigation is needed to grasp the underlying mechanisms and implications of these correlations in a Kosovo context.

**Disclosure of Interest:**

None Declared

